# Stress management in nurses caring for COVID-19 patients: a qualitative content analysis

**DOI:** 10.1186/s40359-022-00834-4

**Published:** 2022-05-17

**Authors:** Mahboobeh Hosseini Moghaddam, Zinat Mohebbi, Banafsheh Tehranineshat

**Affiliations:** 1 Department of Nursing, School of Nursing and Midwifery, Shiraz University of Medical Sciences, Shiraz, Iran; 2 Community Based Psychiatric Care Research Center, School of Nursing and Midwifery, Shiraz University of Medical Sciences, Shiraz, Iran

**Keywords:** Stress, Management, Nurses, COVID-19

## Abstract

**Background:**

Being in the frontline of the battle against COVID-19, nurses need to be capable of stress management to maintain their physical and psychological well-being in the face of a variety of stressors. The present study aims to explore the challenges, strategies, and outcomes of stress management in nurses who face and provide care to COVID-19 patients.

**Methods:**

The present study is a qualitative descriptive work that was conducted in teaching hospitals affiliated with Shiraz University of Medical Sciences, Iran, from June 2020 to March 2021. Sixteen nurses who were in practice in units assigned to COVID-19 patients were selected via purposeful sampling. Data were collected through semi-structured, individual interviews conducted online. The collected data were analyzed using MAXQDA 10 according to the conventional content analysis method suggested by Graneheim and Lundman.

**Results:**

The data collected in the interviews resulted in 14 subcategories under 4 main categories: providing care with uncertainty and anxiety, facing psychological and mental tension, creating a context for support, and experiencing personal-professional growth.

**Conclusions:**

The nurses caring for COVID-19 patients needed the support of their authorities and families to stress management. Providing a supportive environment through crisis management training, providing adequate equipment and manpower, motivating nurses to achieve psychological growth during the pandemic can help them manage stress.

**Supplementary Information:**

The online version contains supplementary material available at 10.1186/s40359-022-00834-4.

## Introduction

Today, COVID-19 is a life-threatening disease all over the world and has become an international concern and a global emergency [1]. Following the spread of the infection to more than 150 countries and its reaching pandemic proportions, the healthcare personnel, especially nurses, have been in the frontline of providing care to the infected [2]. The nature of healthcare professions, nursing in particular, involves working in highly stressful conditions [3]. The results of several studies show that prolonged exposure to stress can cause nurses and other healthcare personnel to suffer such consequences as a reduction in their physical and psychological health, lower job satisfaction, reduced efficiency and quality of care, and an increase in the rate of job burnout [2, 4].

The members of healthcare teams, especially nurses, are exposed to many occupational hazards and experience high levels of stress as a result. According to a study, the nurses who cared for severe acute respiratory syndrome (SARS) patients suffered from high levels of psychological distress [5]. At the height of SARS and Middle East respiratory syndrome coronavirus (MERS-CoV) epidemic, nurses and medical students in Taiwan and Saudi Arabia who cared for the infected showed signs of psychological issues, including anxiety, stress and aggressiveness [6]. Fear and anger were other distressful emotions experienced by nurses who provided care to MERS-CoV patients in Saudi Arabia [7].

The results of recent studies show that the healthcare personnel have experienced high levels of anxiety since the outbreak of the COVID-19 pandemic [1, 8]. The pandemic also affected the psychological health of a considerable number of healthcare professionals in Spain, so much so that their resilience was at risk in case of another wave of the infection. Severe stress was often caused by fear of being infected and infecting one’s loved ones. These difficult conditions resulted in care providers’ lack of empathy with COVID-19 patients and inconsistency in caring for the patients [9]. According to another study in South America, many healthcare professionals experienced lack of personal protective equipment (PPE), including gowns, masks, and face shields, during the pandemic. Concern about lack of equipment and fear of being infected were greater in the personnel who were involved in procedures in which aerosols were produced. Some care providers had to use their gowns and N95 masks several times because there was a shortage of protective equipment [10]. A study by Zhang et al. (2020) in China showed that, in the early stages of caring for COVID-19 patients, nurses were divided between their professional commitment and fear of being infected. The nurses who had been isolated for 1–2 weeks suffered emotional exhaustion; they were able to adapt psychologically after 3–4 weeks of working in isolated wards [11].

According to studies in Iran, the causes of care providers’ heightened anxiety in caring for COVID-19 patients are fear of being infected, the difficulty of controlling the pandemic and lack of medical equipment [1, 12, 13], death anxiety, the little-known nature of the infection, lack of time, spread of bad news, obsessive thoughts and the public’s disregard for preventive measures [14]. Another study in Iran reported administrative issues to be the most significant source of stress for nurses who provide care to COVID-19 patients [15].

Long working hours and work overload, exposure to infection and close contact with COVID-19 patients, the stigma of being a potential carrier of the infection, social media pressures, and increase in the number of death cases lead to fatigue, despair, and helplessness in the nurses and undermine the quality and quantity of nursing care [12]. Other consequences of job fatigue in nurses are absence, delay, job burnout, and concentration disorders, with adverse effects on patient safety [16, 17].

Compared to the other professionals in the healthcare system, nurses spend more time with patients and play a key role in controlling and treating emerging diseases [18]. Research shows that nurses have experienced higher levels of occupational and psychological stress during the COVID-19 pandemic [8, 17]. Nurses’ psychological health correlates with the quality of healthcare services, and occupational stress is one of the most influential factors in the psychological health of this population [19]. Accordingly, it is necessary to evaluate nurses’ mental health and stress management skills in facing and caring for COVID-19 patients and to identify barriers to their stress management during the current pandemic. Considering the above-mentioned points and the fact that the researchers could not find any studies which investigated nurses’ stress management at the height of the COVID-19 pandemic, a qualitative study could provide an in-depth understanding of the subject in question. Accordingly, the present study uses a qualitative approach to explore the challenges, strategies, and outcomes of stress management in nurses who face and provide care to COVID-19 patients.

## Materials and methods

The present study is a qualitative descriptive work of research with a content analysis design which was conducted from June 2020 to March 2021 in teaching hospitals affiliated with Shiraz University of Medical Sciences. The participants of the study were nurses who were in practice in hospitals dedicated to the treatment of COVID-19 patients or back-up hospitals. As the pandemic continued, all the hospitals in Iran had to admit COVID-19 patients; thus, in every hospital, certain wards, including surgical and internal wards, were assigned to the care and treatment of the infected.

In total, 16 nurses who were in practice in special care, internal, surgical and emergency departments were selected via purposeful sampling [20]. Sampling continued until the data was saturated, i.e. no new knowledge could be obtained and the categories were saturated in terms of characteristics and dimensions. The researchers reached data saturation after 14 interviews. However, two more interviews were conducted to verify data saturation. Analysis of the last two interviews did not yield any new codes. The research team and two qualitative research experts examined the codes to verify that the data were saturated.

The inclusion criteria were: having a bachelor’s degree in nursing, having at least six months’ experience of full-time practice as a clinical nurse, having at least 2 weeks’ experience of caring for COVID-19 patients, and willingness to share one’s experiences with the researchers. The subjects who were not willing to be interviewed or continue their participation in the study were excluded. Data were collected through 16 in-depth, semi-structured, individual interviews which were conducted by the third author.

The participants were interviewed by video calls with “WhatsApp”, in workplace (one of the classes at school of nursing and midwifery), after arrangements about the time of the interviews had been made with them. Each interview lasted from 40 to 60 min and began with a general question: “Can you describe your experiences of a work shift in which you faced or cared for a COVID-19 patient?” Subsequently, the interviewer asked more specific questions: “What are your experiences of the challenges and barriers to stress management when you faced and cared for COVID-19 patients?”, “What factors improve stress management when nurses face and care for COVID-19 patients?”, “What factors undermine stress management when nurses face and care for COVID-19 patients?”, “How did you feel when/if you could not manage your stress when you faced and cared for COVID-19 patients?”, “What stress management strategies can help nurses who face and care for COVID-19 patients?” and “What are the outcomes of stress management for nurses who face and care for COVID-19 patients?” Moreover, follow-up questions were asked to obtain more details about the objective of the study (Additional file 1: Interview Guide). The participants' voices were recorded using a Sony Voice Recorder ICD-TX650.

The present study used the conventional content analysis method suggested by Graneheim and Lundman (2004). Frequently used in studies related to nursing, this method allows for collecting new, rich information and mental analysis of the content of textual data through systematic categorization, codification and theme making or developing known paradigms [21, 22]. Each interview was transcribed in full immediately after it was completed. To immerse in the data and obtain a general idea of the participants’ answers, the researchers read the transcripts several times. Words, phrases and paragraphs which carried significance with regard to the challenges, strategies and outcomes of stress management in the COVID-19 crisis were selected as meaning units. Similar initial codes were classified into broader categories based on their similarities and differences and categories were thus developed. To ensure that the codes were consistent, the researchers reviewed the categories and compared them to the data again. Afterward, through deep and accurate contemplation and comparison of the categories against each other, the themes emerged [23]. The data were organized using MAXQDA 2010 distributed by VERBI.

The trustworthiness of the collected data was ensured using Lincoln and Guba’s criteria [24]. Credibility was achieved through prolonged engagement with the data, member checking, peer debriefing, maximum variation sampling, and searching for contrasting evidence. To ensure dependability and confirmability, the researchers relied on audit trial which consists of using proper techniques to conduct interviews, making accurate transcripts and having the data reviewed by one’s co-researchers. To enhance the transferability of the results, the researchers provided accurate and thorough descriptions of the subject under study, the participants’ characteristic, methods of data collection and analysis, along with documented examples of the participants’ statements [25].

## Results

The participants’ ages ranged from 25 to 48 years, with the mean being 34.93 years. The majority of the participants were female (Table 1). Analyses of the data resulted in 510 initial codes, 14 subcategories and four main categories, namely: providing care with uncertainty and anxiety, facing psychological and mental tension, creating a context for support, and experiencing personal-professional growth (Table 2). Table 3 presents an example of meaning units, coding, and development of sub-categories and categories. Providing care with uncertainty and anxiety and facing psychological and mental tension were the challenges which the nurses in the present study experienced when they faced and cared for patients with COVID-19.

**Table 1:** The demographic characteristics of the participants

Participants	Age	Gender	Marital status	Position	Work experience (years)	Ward
P1	35	Female	Married	Head nurse	12	ICU
P2	36	Female	Single	Staff nurse	10	Internal
P3	31	Female	Single	Staff nurse	9	ICU
P4	29	Female	Single	Staff nurse	4	ICU
P5	25	Male	Single	Staff nurse	2	ICU
P6	37	Female	Married	Staff nurse	12	ICU
P7	35	Female	Single	Staff nurse	11	Thorax surgery
P8	47	Female	Married	Head nurse	20	Thorax surgery
P9	30	Male	Single	Staff nurse	8	Emergency
P10	29	Female	Married	Staff nurse	7	Emergency
P11	45	Female	Married	Staff nurse	18	Internal
P12	38	Female	Single	Staff nurse	9	ICU
P13	28	Female	Single	Staff nurse	5	ICU
P14	34	Male	Married	Staff nurse	6	Thorax surgery
P15	32	Female	Single	Staff nurse	8	ICU
P16	48	Female	Married	Staff nurse	22	ICU

**Table 2:** The categories and sub-categories of this study

Categories	Sub-categories	Selective coding
Providing care with uncertainty and anxiety	Providing care as a professional duty	Providing care voluntarily Feeling committed to one’s job as a care-provider Having a sense of responsibility in providing care to COVID-19 patients
	Concern over transmitting the infection to one’s family	Concern over hugging one’s child Concern over eating with one’s family Nightmares about giving the infection to one’s family Staying away from one’s mother Not sleeping next to one’s child
	Fear of the unknown aspects of the disease	Fear of ignorance about how the infection is transmitted Fear of ignorance about the symptoms of the infection Fear of ignorance about the fatality of the infection Fear of ignorance about how to disinfect medical equipment Fear of ignorance about treatment of the infection
	Concern over making wrong decisions	Concern over volunteering to work in wards for COVID-19 patients Hesitation about trying to achieve professional development during the pandemic Hesitation about trying to achieve professional goals during the pandemic
	Families’ insistence on quitting one’s job	Father’s disapproval of continuing one’s job Spouse’s insistence on quitting one’s job Relatives’ insistence on quitting one’s job Mother’s insistence on quitting one’s job
Facing psychological and mental tension	Working in difficult conditions	Witnessing the deaths of one’s friends Witnessing the fear and anxiety of one’s colleagues Witnessing the loud objection of one’s colleagues who are not willing to work in wards for COVID-19 patients Having shortness of breath when one is wearing a mask Not having enough time to eat Not having enough time to drink anything Suffering dehydration Getting a sore nose as a result of wearing masks for long periods
	Lack of personal protective equipment	Lack of N95 masks Lack of gowns Lack of face shields
	Feeling rejected	Unfriendly looks from one’s friends and acquaintances Fewer family visits Fewer interactions with one’s family and relatives Fewer interactions with one’s friends Being avoided by one’s relatives outside home
Creating a context of support	Proper intradepartmental management	Satisfactory distribution of tasks among the personnel Distribution of tasks among the personnel based on their emotional traits Equitable distribution of PPE Satisfactory job rotation Designation of hours for the personnel to visit a counselor Participation of the personnel in inter-ward decision making Monitoring infection control in the ward
	Support of the authorities	Rewarding the personnel Granting official appreciation cards Granting time-off awards Reducing the personnel’s obligatory working hours Reducing the shifts of the personnel in COVID-19 wards Organizing question and answer sessions Addressing the personnel’s problems and issues Giving priority to job applicants who volunteer to work in COVID-19 wards
	Effective communication skills	Empathizing Visiting the personnel infected with COVID-19 Giving positive feedback to one’s colleagues Raising one’s colleagues’ spirits
Experiencing personal-professional growth	Improved learning	Sharing one’s experiences Becoming acquainted with the symptoms of emerging diseases Learning how to care for COVID-19 patients Learning how to properly use PPE Developing one’s time management skills
	Perception of positive feelings at the end of a crisis	Development of friendly relationships between the personnel Obtaining a sense of achievement Improving the social image of nurses Strengthening relationships between the personnel Developing a sense of self-efficacy Developing self-sufficiency
	Self-transformation	Increased flexibility Approaching time spent with one’s family Enhanced self-organization Stronger spirituality

**Table 3:** An example of coding and development of sub-categories and categories

Meaning units	Coding	Sub category	Category
“When your shift is over, you can barely breathe; with a high PaCO2, it’s hard to breathe … it’s hard to eat in this coverall, you can’t drink any water through your shift … the fatigue …. Working in such conditions won’t let you manage stress…” (P12) “The annoying thought of having to put on gear that’s a torture …” (P16)	Having shortness of breath when one is wearing a mask Not having enough time to eat Not having enough time to drink anything Listlessness Increased difficulty of having to work in protective gear	Working in difficult conditions	Facing psychological and mental tension
“When the epidemic started, we didn’t have access to special gear for COVID-19 protection and had to care for the infected with minimum equipment, in regular masks and uniforms …” (P5)	Lack of special protective gear for caring for COVID-19 patients Lack of gowns Lack of face shields	Lack of personal protective equipment	
“When I got on the hospital shuttle, I felt so nervous. All the other staff that didn’t work in COVID-19 units would protest and tell the driver that he shouldn’t let me get on board … I should get off ….” (P 7)	Objection of nurses from other wards to the boarding of nurses from COVID-19 wards Being rejected by one’s colleagues	Feeling rejected	

### Providing care with uncertainty and anxiety

One of the findings of the present study was providing care with uncertainty and anxiety. This category consisted of the subcategories of providing care as a professional duty, concern over transmitting the infection to one’s family, fear of the unknown aspects of the disease, and concern over making wrong decisions.

#### Providing care as a professional duty

According to the participants’ experiences, despite a lack of facilities and personal protective equipment, there was a dominant sense of commitment among nurses to perform their professional duties in the COVID-19 pandemic. From the nurses’ point of view, working in difficult and dangerous conditions is part of a nurse’s job which must be maintained during the pandemic of an emerging disease.

“… anyway, it’s my job to give care in all circumstances and I had to do it at that time …” (P1).

According to another participant:

“…I volunteered to work in the COVID-19 ward … I thought to myself, ‘This is my job and my duty …” (P15).

#### Concern over transmitting the infection to one’s family

Despite their commitment to perform their professional duties, the nurses in the present study were worried about transmitting the infection to their families and their concern acted as a barrier to their stress management. The participants also mentioned that when their colleagues were infected, the fear of contracting the disease in the near future was a barrier to their stress management.

“… I was really worried that I could be the source of infection to my 60-year-old parents …” (P6).

#### Fear of the unknown aspects of the disease

One of the major barriers to stress management in the face of COVID-19 was lack of knowledge about the nature of the infection. Fear of the personnel arising from the unknown aspects of the disease was a main source of stress.

“… We didn’t know anything about it. The disease was completely unknown to us …” (P 3).

#### Concern over making wrong decisions

Some of the nurses were working in COVID-19 units voluntarily; however, they admitted that they had felt doubtful about their decision to provide voluntary care to COVID-19 patients in the first few days.

“… I kept wondering if my decision was right or not …” (P 4).

Another nurse stated that:

“…Maybe the decision I made was a step to fulfill my personal commitment …” (P 2).

### Facing psychological and mental tension

Many of the nurses who were working in COVID-19 units declared that they had experienced a variety of psychological issues during their practice in these units which prevented them from managing their stress. This category consisted of the sub-categories of families’ insistence on quitting one’s job, working in difficult conditions, lack of personal protective equipment, and feeling rejected.

#### Families’ insistence on quitting one’s job

One of the sources of psychological tension for the nurses was the insistence of some of their family members that they should quit their profession as nurses during the pandemic.

“…My dad was strongly against me staying in this profession at those critical times …” (P 3).

#### Working in difficult conditions

Many of the participants described working in personal protective gear as very difficult. They also referred to work fatigue and physical exhaustion due to work overload as barriers to stress management.

“…When your shift is over, you can barely breathe; with a high PaCO2, it’s hard to breathe … it’s hard to eat in this coverall, you can’t drink any water through your shift … the fatigue …. Working in such conditions won’t let you manage stress…” (P 12).

According to another nurse:

“…Our workload has increased at these stressful times caused by the pandemic; my life and my family’s life have been affected … it’s not easy to work with all this protective gear on … Such things as fogging of my face shield interfered with my job … I was exhausted, both physically and emotionally…” (P16).

#### Lack of personal protective equipment

The participants stated that one of the major barriers to stress management was lack of personal protective equipment and coveralls, especially in the first few days of the pandemic. According to one of the nurses:

“…When the epidemic started, we didn’t have access to special gear for COVID-19 protection and had to care for the infected with minimum equipment, in regular masks and uniforms …” (P 5).

#### Feeling rejected

Another barrier to stress management in COVID-19 units was being treated inappropriately and rejected by the personnel in non-COVID-19 units. One of the participating nurses stated that:

“…When I got on the hospital shuttle, I felt so nervous. All the other staff that didn’t work in COVID-19 units would protest and tell the driver that he shouldn’t let me get on board … I should get off ….” (P 7).

Some of the participants had experienced rejection by their family members and relatives, which made it more difficult for them to manage stress.

“…Once, when one of my relatives saw me, she took her son’s hand and walked away from me …” (P 3).

### Creating a context of support

In the present study, the participants believed that creating a context of support is an important stress management strategy in facing and caring for COVID-19 patients. This category consists of the subcategories of proper intradepartmental management, support of the authorities, and effective communication skills.

#### Proper intradepartmental management

The participants’ experiences showed that proper intradepartmental management, e.g. planning according to the personnel’s conditions, replacing COVID-19 personnel with volunteers, avoiding discrimination, playing soft music in the units, and using effective interpersonal communication skills, including empathy, humor and spreading positive thinking, can contribute to the personnel’s stress management. During the COVID-19 crisis, the unit managers tried to make plans according to the personnel’s conditions in order to reduce the personnel’s stress.

“…The husband of one of my staff here could spend two weeks a month with his wife …. I arranged her shifts so she didn’t have to work or worked less when her husband was with her so she wouldn’t be so worried about infecting her husband …” (P 1).

To reduce nurses’ direct contact with COVID-19 patients, the unit managers put the overstressed nurses in charge of recording patients’ history in their files in order to reduce stress in them.

“…One of my staff was stressed out …. I made her the shift supervisor so she would be busy with the patients’ files and have less direct contact with the patients …” (P 8).

#### Support of the authorities

In order to support the nurses by helping them manage their stress, the unit and hospital authorities arranged certain hours for the nurses to meet with the hospital counselor or for the counselor to see the nurses.

“…Whenever the personnel felt they were suffering from psychological tension and needed counseling, they could visit the hospital counselors ….” (P 11).

Another strategy used by the authorities to help the nurses manage their stress was setting up workshops to inform the nurses about the pathophysiology of COVID-19, how the infection is transmitted, the correct use of personal protective equipment, and regimens that boost the immune system. This information proved very influential in reducing stress in the nurses.

“…We didn’t have any preparation from before; education is really important; once we learned more about the disease, we could protect ourselves better …. We were less stressed …” (P 12).

According to the participants, continuing education was integral to enabling them to manage the stress caused by being in contact with COVID-19 patients. Even though the presence of the supervisors and the other members of the treatment team in the environment could communicate a sense of support and hope to the nurses, some of the supervisors refused to be present in the places where the patients were being cared for.

“…Our supervisor won’t even enter the unit to see for herself what kind of issues we are dealing with here …” (P 9).

#### Effective communication skills

The head nurses’ use of effective communication skills in their interactions with the staff was found to be a successful approach to stress management in those critical times. In addition, through empathy, the unit personnel tried to connect to their colleagues’ inner worlds and have a mutual understanding of their emotions and concerns, thereby coping with the stress caused by the COVID-19 crisis. The participants stated that in an empathetic relationship, they could experience a sense of support.

Showing appreciation and giving rewards were found to be effective stress management strategies which could raise the personnel’s spirits and improve interpersonal relationships. According to one of the participants:

“…One of the main issues for the nurses who are doing a good job in these units is not receiving any appreciation or rewards. There is a limit to any person’s tolerance. Some turn to spirituality for strength, but not everyone is spiritual. In short, there is a lack of appreciation …” (P 5).

Other behaviors which contributed to the nurses’ stress management were exchanging friendly banters with each other, spreading positive thinking, and remaining optimistic about the future.

### Experiencing personal-professional growth

The participants referred to experiencing personal-professional growth as one of the outcomes of working in the very difficult conditions created by the spread of COVID-19. Caring for COVID-19 patients was a constructive experience which could prove instrumental in coping with problems in the future. Making an effort to use various stress management skills in the face of COVID-19 improved nurses’ empowerment and gave them a chance for personal growth. Compared to the time before the emergence of COVID-19, nurses can focus on their problems better and manage stress and stressors more effectively. This category consists of the subcategories of improved learning, perception of positive feelings at the end of a crisis, and self-transformation.

#### Improved learning

The participants believed that, despite all the difficulties, working during the pandemic had resulted in their gaining useful knowledge. Their experiences showed that providing care during the COVID-19 pandemic had helped them develop time management skills, learn to make optimal use of the available equipment and deal with deficiencies, improve their medication knowledge, increase their knowledge of emerging diseases, and learn to make effective use of infection control strategies.

“…I had read a few things about emerging diseases, but working in this pandemic has given me the chance to gain hands-on experience of caring for these patients …” (P 7).

#### Perception of positive feelings at the end of a crisis

While providing care to COVID-19 patients, the participants had experienced such positive feelings and emotions as elevated self-confidence, personal satisfaction, the opportunity to prove their competence, and the good feeling of overcoming the difficulties and challenges of caring for COVID-19 patients.

“…Working in these conditions created a positive sense of being useful to others in me … which made me feel happy and lively and physical fatigue could not take away my happiness …” (P 6).

Many of the participants mentioned feeling good about solving problems and happiness about serving one’s fellowmen to be among other outcomes of working in the COVID-19 crisis.

“…Having been put on the path to serve my fellowmen was the positive feeling that I experienced …” (P 4).

Another participant stated:

“…I feel happy now that I could live through the problems I had at the time …” (P 12).

#### Self-transformation

The results of the study showed that the experience of caring for COVID-19 patients in difficult conditions had strengthened relationships between colleagues and between friends at work. Many of the participants had come to have higher regard for the meaning and value of their lives and what they had. The participants compared working in the COVID-19 crisis to fighting in a war which had transformed them by making them braver in the face of critical conditions and more empowered in managing difficult times.

“…Working during the current pandemic has helped me discover my abilities …. Now I know how to act in war conditions …” (P 7).

## Discussion

The present study was conducted to identify the challenges, strategies and outcomes of stress management for nurses who come into contact with and provide care to COVID-19 patients. Analyses of the collected data showed that the nurses’ experiences could be classified into four categories: providing care with uncertainty and anxiety, facing psychological and mental tension, creating a context for support, and experiencing personal-professional growth.

One of the findings of the present study was nurses’ feelings of uncertainty and anxiety in providing care. The nurses were worried about transmitting the infection to their family members, were not adequately aware of the nature of the disease, and were in fear of making wrong decisions about caring for COVID-19 patients; yet, they deemed it their professional duty to provide care to those patients. In another study, the nurses were worried about facing patients with COVID-19, contracting the infection and transmitting it to their loved ones. The nurses were also concerned about the unknown aspects of the disease and its potentially dangerous nature, uncertainty about the end of the pandemic, and the impact of the pandemic on their job security and other working conditions [26]. Another study reported that the healthcare personnel suffered psychosocial issues, e.g. depression, anxiety, and stress, as a result of having inadequate knowledge about COVID-19, continuously caring for the infected, work overload, frequent exposure to distressful events, such as death, and fear of infecting their families [27]. In yet another study, the nurses suffered from stress due to caring for the infected and work overload, which resulted in their experiencing burnout, emotional fatigue, and despair [28]. De Kock et al. found that, the COVID-19 crisis had an impact on the nurses’ mental health and increased their anxiety, stress, and sleep difficulties. The occupational, psychosocial, and environmental risk factors associated with adverse mental health outcomes during the COVID-19 pandemic identified through their study [29]. The frontline healthcare workers commonly reported increased workloads, which impacted on their psychosocial health. The healthcare workers reported increased hours and weekend shifts, additional time taken to manage PPE and staff shortages as frequent sources of stress. However, because of staff shortages, some nurses described feeling guilty for taking time off to rest [30].

Several studies suggest long-distance psychological interventions, including video conferencing, online programs, applications, and even telephone calls, to help care providers cope with their anxiety [31, 32]. In China, Internet-based programs are used to screen and identify individuals who are prone to psychological disorders and to provide self-learning protocols and guidelines to the healthcare personnel and the general public in the form of videos and pamphlets [33]. During a pandemic, digital interventions should be used to address anxiety, depression, self-torture, and suicide, as well as to gather information, perform triage and introduce medical interventions via online applications or phone calls and text messaging for those with limited access to digital sources [34, 35]. Measures, such as workshops and counseling with a psychologist, should be taken to decrease stress and anxiety in nurses who are directly involved in caring for COVID-19 patients as prolonged anxiety can lead to psychological distress, depression and other psychological issues.

Another finding of the present study was nurses’ exposure to mental and psychological tension which originated from the insistence of their families that they should quit their jobs, feeling rejected, witnessing the death of their colleagues and friends, fear of infecting their families, lack of medical equipment, and fatigue. According to one study, the major contributory factors in the psychological pain of nurses, doctors, therapists and other healthcare professionals were the emotional efforts and physical fatigue that they experienced in caring for COVID-19 patients whose conditions were deteriorating quickly. Seeing and caring for colleagues who were severely infected or lost their lives to COVID-19, lack of ventilators and other medical equipment needed to care for patients in critical conditions, and anxiety due to new clinical roles and work overload were other causes of psychological distress [36].

As a result of social stigmatization, the nurses suffered from social dejection and loneliness. They preferred to be isolated because they were afraid that they might transmit the infection to others [37]. Another study reported that frequent changes in the hospital policies, routines, and guidelines which were updated confused the nurses [38]. In another study, the participants reported work overload as a result of lack of medical protective equipment: the nurse managers had to reduce the number of care providers to ensure that all the personnel had access to protective equipment by conserving it. Moreover, to avoid going to break rooms during working hours, the healthcare professionals did not eat anything, and they experienced severe emotional distress and grief if their patients passed away [39]. During the COVID-19 pandemic, nurses were exposed to so many psychological issues that they sometimes had to resign [40].

According to Maslow's hierarchy of needs, physiological and safety needs are two of the basic and essential needs of mankind. If these needs are satisfied in them, nurses can experience self-esteem, love and self-actualization and, consequently, meet other individuals’ needs without feelings of exhaustion or burnout. Thus, authorities and governments should decrease the physical and mental pressure caused by the COVID-19 pandemic by creating an appropriate work environment, supplying adequate PPE, managing work shifts effectively, and considering the physical and mental health of nurses [12]. Support interventions, including informing the public about the pandemic and self-quarantine, providing enough resources to meet the basic needs of nurses in quarantine, and giving nurses easy access to social media so that they can be in touch with their friends and families can encourage nurses to stay in their jobs [37]. Also, counseling the personnel and preparing them for the challenges in their profession and giving a clear picture of what they are going to face can prove helpful [41].

Research findings show that mental health interventions for the time of the pandemic, including crisis management, education in resilience, improvement in quality of life and life satisfaction, confrontational strategies, education via guidelines and protocols, screening programs and long-distance Internet-based interventions, are effective methods to manage the psychological consequences of the pandemic for nurses [42, 43]. Strategic support and planning across the country and arranging for psychological first aid during crises which are potentially offered by telemedicine are among the interventions which must be employed. In addition, comprehensive plans should be developed to reduce mental pressure on nurses through prevention and intervention, e.g. screening and purposeful visits, to avoid further mental health issues in this population [44, 45]. Educational programs about death anxiety can result in better nursing care in critical situations. Educational workshops are effective in reducing death anxiety [14]. Giving nurses active guidance and motivating them to achieve psychological growth during the pandemic can contribute to their psychological adaptation [46].

Another finding of the present study was the role of creating a context of support. The participants’ experiences showed that intradepartmental management, support of the authorities, and effective communication skills are effective ways to support nurses in the stressful conditions caused by the pandemic. A context of support will reduce nurses’ anxiety and help them continue to provide quality healthcare services in the crisis. According to one study, personal resilience protects nurses from anxiety by enabling them to adapt to irritating, stressful conditions. In addition, public support for nurses plays a significant role in their achieving positive emotional states in stressful times, such as the outbreak of a disease [47]. In another study, the nurses practiced self-isolation as a protection strategy [38]. The nurses believed that one of the greatest challenges in their workplace during the pandemic was lack of a crisis management plan on the management and ministry of healthcare levels [48]. In Billings et al. study working with coworkers during the pandemic was considered to provide an important source of mutual support and opportunities to learn from each other. The employees reported feeling supported by their organizations when there was a clear alliance and shared decision making between senior managers and frontline healthcare workers [30].

The experiences of the nurses and doctors in another study showed that one way to prevent the physical and emotional exhaustion caused by working in special care units is using educational interventions to develop resilience in the personnel. Also, regular and systematic educational programs designed to improve the healthcare personnel’s preparation and efficacy in crisis management seemed essential [49]. Healthcare professionals were also found to rely on self-management mechanisms. They preferred to focus on their work rather than listen to rumors about COVID-19. Some of the other personnel engaged in relaxation activities, including seeing movies, taking showers, and reading. They also stressed the significance of good nutrition and adequate rest and sleep [39].

Moreover, stress management approaches, including relaxation, biofeedback, cognitive strategies, interventions focused on eliminating or decreasing stressful working conditions, such as job redefinition and clear job description, formation of joint personnel committees, and increasing the personnel’s participation in organizational decisions are essential to controlling stressful conditions [50]. One support strategy for the personnel in the frontline of caring for patients with COVID-19 is providing them with online psychological support services which enable the personnel to express their needs. Access to mental health care is possible through telemedicine managed by mental health experts, mobile phone applications, online sources, and virtual peer support. Online psychological care networks are useful in dealing with the stress and concerns of healthcare professionals and their families and relatives and other individuals [51]. One study classifies nurse managers’ experiences of management of the nursing workforce during the COVID-19 crisis into management of workforce recruitment (volunteers and non-volunteers) and management of workforce (via motivational measures and psychological support). Successful crisis management depends on using flexible and situation-based principles of management toward recruiting adequate workforce and retaining the current workforce [52]. By creating a pleasant work environment, encouraging colleague support, and raising trust in the personnel, nurse managers can improve nurses’ stress management skills. Nurse managers play a key role in the promotion of inter-professional relationships in clinical environments, which can contribute to nurses’ mental health [11]. Providing a supportive environment through crisis management training, providing adequate equipment and manpower, and developing resilience skills in staff helps nurses manage stress.

Another finding of the present study was the nurses’ experiencing personal and professional growth. This theme consisted of the subcategories of improved learning, perception of positive feelings at the end of a crisis, and self-transformation. According to a study, one of the psychological experiences of the nurses who cared for COVID-19 patients was perception of growth under pressure and appreciation of life. The nurses declared that, in the pandemic, the picture of the nursing profession had improved, nurses had realized their potential, and nurses’ ability to cope with difficulties in life had elevated [46]. Another study reported an increase in nurses’ self- esteem and sense of responsibility during the pandemic [39]. One of the significant experiences of new nurses was learning during the pandemic. The nurses stated that caring for COVID-19 patients gave them a chance to develop their clinical skills, gain hands-on clinical experience, and learn how to adapt to critical conditions. They also learned methods of protecting themselves, using the equipment needed in caring for COVID-19 patients, time management, and providing care in the shortest possible time to minimize contact with the infected [53]. Another study reported an increase in nurses’ awareness about the value of the nursing profession and their sense of achievement and pride. Moreover, the nurses had a better perception of their professional and social identity and established stronger relationships with their colleagues. Many of the nurses had a greater appreciation of life and everything which they had. The pandemic had given them a chance to realize their full potential, which, in turn, increased their self-confidence. In addition, by self-reflection, the nurses discovered their weaknesses and tried to develop their knowledge and skills. They appreciated the support of their friends, colleagues and the society and developed a more positive perspective on the significance of life and family health [54].

Appreciating life and experiencing increased self-power are the characteristics of post-traumatic growth which may emerge in individuals following a stressful experience. As a result of cognitive reconstruction after trauma, individuals experience personal vulnerability and come to believe that they are not capable of predicting and controlling certain events in life. In response to the unstable nature of life, individuals reconsider the little things in life which used to be important or unimportant to them, which in turn, results in their changing their priorities in life and having a deeper apperception of life [55]. The results of studies show that projecting a positive image of the nursing profession in the society helps nurses find their potential, improves nurses’ professional knowledge and skills in the epidemic of emerging diseases, and increases nurses’ awareness of their personal and professional growth and professional identity by extension.

As the main strength of this study, we conducted one of the qualitative studies that explored strategies and outcomes of stress management based on experiences of the nurses who provide directly care to COVID-19 patients. One of the limitations of the present study was the fact that, because of the COVID-19 pandemic, the interviews were not conducted on a face-to-face basis. However, the researchers tried to have a better interaction with the participants by making video calls to them. Another limitation of the study was that the interviews were conducted individually. Focus group interviews allow for the collection of richer information.

## Conclusion

Despite the fact that the nurses in the present study regarded caring for COVID-19 patients as their professional duty, they needed the support of their superiors, families, and friends. Authorities can create an effective context of support through proper intradepartmental management, providing adequate equipment, giving positive feedback, and organizing continuing workshops. In addition, effective communication skills, receiving positive feedback and easy access to counselors can contribute to nurses’ stress management and adaptation. The outcomes of stress management on the part of nurses were found to include understanding the meaning of life and experiencing personal and professional growth. It is suggested that future studies address the causes of barriers to nurses’ stress management in the COVID-19 crisis and the strengths and weaknesses of the healthcare system in dealing with this pandemic. It is also suggested that future research address the lived experiences of change in the performance and behaviors of care providers who care for COVID-19 patients.

## Supplementary Information

**Additional file 1.** Interview guide.

## Data Availability

The dataset generated and/or analysed during the current study are not publicly available due to promises of participant anonymity and confidentiality but are available from the corresponding author on reasonable request.

## Abbreviations

COVID-19: Coronavirus infection disease-19
SARS: Severe Acute Respiratory Syndrome
MERS-CoV: Middle East respiratory syndrome coronavirus
PPE: Personal protective equipment
