# Supplementary material for: Stress management in nurses caring for COVID-19 patients: a qualitative content analysis
Source: BMC Psychol. 2022 May 17;10:124. doi: 10.1186/s40359-022-00834-4 (PMC9112256; doi:10.1186/s40359-022-00834-4)
Supplement: Supplementary file 1 — Additional file 1. Interview guide. [file 40359_2022_834_MOESM1_ESM.docx]

Thank you for participating in the present study. The objective of the study is to determine the challenges, strategies, and outcomes of stress management by nurses who face and care for COVID-19 patients. You are going to be asked a few questions about the subject of the study. If you find a question ambiguous, please ask us for clarification. All the information collected during the interview will be confidential and only the research team will have access to it. The interview will be audio recorded to ensure that the researchers will not miss any significant points. The recorded interview and your personal information will be treated as confidential. Your interview will be assigned a code. Do you consent to the recording of your interview with a voice recorder?

**Personal information**

Name:

Gender:

Marital status:

Professional position:

Work experience:

Ward of practice:

**General question**

Can you describe your experiences of a work shift in which you faced or cared for a COVID-19 patient?

**Specific questions**

What are your experiences of the challenges and barriers to stress management when you faced and cared for COVID-19 patients?

What factors improve stress management when nurses face and care for COVID-19 patients?

What factors undermine stress management when nurses face and care for COVID-19 patients?

How did you feel when/if you could not manage your stress when you faced and cared for COVID-19 patients?

What stress management strategies can help nurses who face and care for COVID-19 patients?

What are the outcomes of stress management for nurses who face and care for COVID-19 patients?
